# Filum terminale transection in pediatric SCIWORA with tight filum terminale: a case series and literature review

**DOI:** 10.3389/fped.2025.1723547

**Published:** 2025-12-29

**Authors:** Yue Yang, Yanfei Wang, Wenwen Tang, Mengyan Yu, Huangyi Fang, Hansong Sheng, Gang Shen

**Affiliations:** 1 Department of Pediatric Neurosurgery, Women and Children’s Hospital of Ningbo University, Ningbo, China; 2 Ningbo Rehabilitation Hospital, Ningbo, China; 3 Department of Neurosurgery, The Second Affiliated Hospital of Wenzhou Medical University, Wenzhou, China

**Keywords:** SCIWORA, filum terminale, pediatric, spinal cord injury, surgery

## Abstract

**Purpose:**

To investigate the role of tight filum terminale (TFT) in pediatric spinal cord injury without radiographic abnormality (SCIWORA) following low-energy trauma and to evaluate the efficacy of minimal invasive interlaminar approach (MIIA) for filum terminale transection in treating these patients. The study aims to determine whether early surgical intervention can improve neurological outcomes in this specific patient population.

**Methods:**

This retrospective case series included four pediatric patients with SCIWORA and concurrent TFT treated at Women and Children's Hospital of Ningbo University from December 2022 to May 2024. The patients underwent MIIA for filum terminale transection. We retrospectively analyzed the medical records, clinical courses, presentations, and treatment strategies for these patients.

**Results:**

All patients in this case series showed evidence of TFT following low-energy trauma. All patients underwent MIIA for filum terminale transection and intraspinal canal exploration due to progressive neurological impairment. None had received steroid treatment. Postoperatively, none experienced further neurological deterioration or complications. Two patients achieved complete resolution of preoperative symptoms within three months, one showed significant neurological improvement, and one had stable neurological status without further worsening.

**Conclusion:**

TFT might be the etiology of SCIWORA in children after suffering from low-energy injuries. Performing filum terminale transection as early as possible after the occurrence of SCIWORA complicated by tight filum terminale in children might be beneficial for relieving the state of spinal cord ischemia and hypoxia caused by longitudinal traction of the spinal cord as early as possible and facilitating the recovery of neurological injuries.

## Introduction

SCIWORA refers to clinical manifestations of spinal cord injury in patients after trauma, without radiographic abnormalities such as radiography or computed tomography (1). SCIWORA was first reported by Pang and Wilberger in 1982 and the term has been used ever since (2). The incidence of SCIWORA is higher in children, accounting for approximately 6%–19% of all pediatric spinal cord injuries (3). This may be attributed to the unique and inherent anatomic malleability of the pediatric spine compared to adults (4). The onset of pediatric SCIWORA is commonly associated with high-energy injuries in traffic accidents and sports competitions. There are also documented cases of SCIWORA resulting from minor trauma during dance activities (5). In recent years, relevant literature has suggested that TFT may be a contributing factor to SCIWORA in pediatric patients following minor trauma, playing a significant role in its pathogenesis (6).

MIIA for filum terminale transection via a microsurgical procedure is commonly used to treat tethered cord syndrome (TCS) caused by tight, thickened, or fat-infiltrated filum terminale. Due to the relatively low surgical difficulty, minimal incision size, and low incidence of complications associated with filum terminale transection, it can significantly improve neurological and functional impairments caused by TCS (7). Some scholars suggest that, given the risk of neurological impairment associated with conservative treatment and the favorable safety profile of MIIA for filum terminale transection, prophylactic surgery based on radiological findings should be considered for patients, regardless of their preoperative symptoms (8). Currently, there is a scarcity of literature reporting on the postoperative efficacy of MIIA for filum terminale transection in pediatric patients with SCIWORA and concomitant tight filum terminale. To address this gap in the literature, this study presents a case series of four pediatric patients with SCIWORA and concomitant tight filum terminale who underwent filum terminale transection at Women and Children's Hospital of Ningbo University. The treatment processes and postoperative outcomes of these patients are detailed in the following sections.

## Materials and methods

The present study included four pediatric patients with SCIWORA complicated by tight filum terminale who received treatment at our center from December 2022 to May 2024. We retrospectively analyzed the medical records, clinical courses, presentations, and treatment strategies for these patients (Table 1). The pediatric patients included in this study were consecutive cases admitted to our center. The inclusion criteria were as follows: patients met the clinical diagnostic criteria for SCIWORA, were aged ≤18 years, and had lumbosacral spine MRI findings suggestive of a possible tight filum terminale (MRI revealed a fat-infiltrated filum terminale or a thickened filum terminale; prone-position lumbosacral spine MRI showed thickening and dorsal deviation of the filum terminale). All patients underwent intraspinal canal exploration and MIIA for FTT treatment and had complete follow-up data. The exclusion criteria included the presence of other spinal cord-related diseases such as spinal cord tumors, spinal deformities, and myelitis, as well as surgical contraindications such as coagulation disorders and severe infections.

**Table 1:** Case characteristics and treatment plans.

Case	Age	Clinical presentation	MRI findings	Treatment	Pathology	Follow-up	Prognosis
1	5 years	Lower limb weakness (grade 0), partial sensory loss, fecal incontinence, urinary retention	Spinal cord edema, thickened filum terminale	MIIA for FTT; intraspinal canal exploration	Fibrous degeneration	6 m	Normal bladder/bowel function; lower limb strength grade 4
2	7 months	Lower limb weakness (grade 0), decreased muscle tone, urinary retention	Fat-infiltrated filum terminale, bladder distension	MIIA for FTT; intraspinal canal exploration	Fat-infiltrated filum terminale	2 m	Normal limb movement; bladder function restored
3	11 years	Urinary retention, constipation	Thickened and dorsally deviated filum terminale	MIIA for FTT; intraspinal canal exploration	Fibrous degeneration	3 m	Normal urinary and bowel function
4	3 years	Lower limb paralysis (Grade 0), sensory loss, urinary retention	Spinal cord edema, thickened and dorsally deviated filum terminale	MIIA for FTT; intraspinal canal exploration	Fibrous degeneration	5 m	No significant motor/sensory improvement; persistent urinary retention; spinal cord atrophy on MRI

MIIA, minimal invasive interlaminar approach; FTT, filum terminale transection.

## Case presentation and results

**Figure 1:**
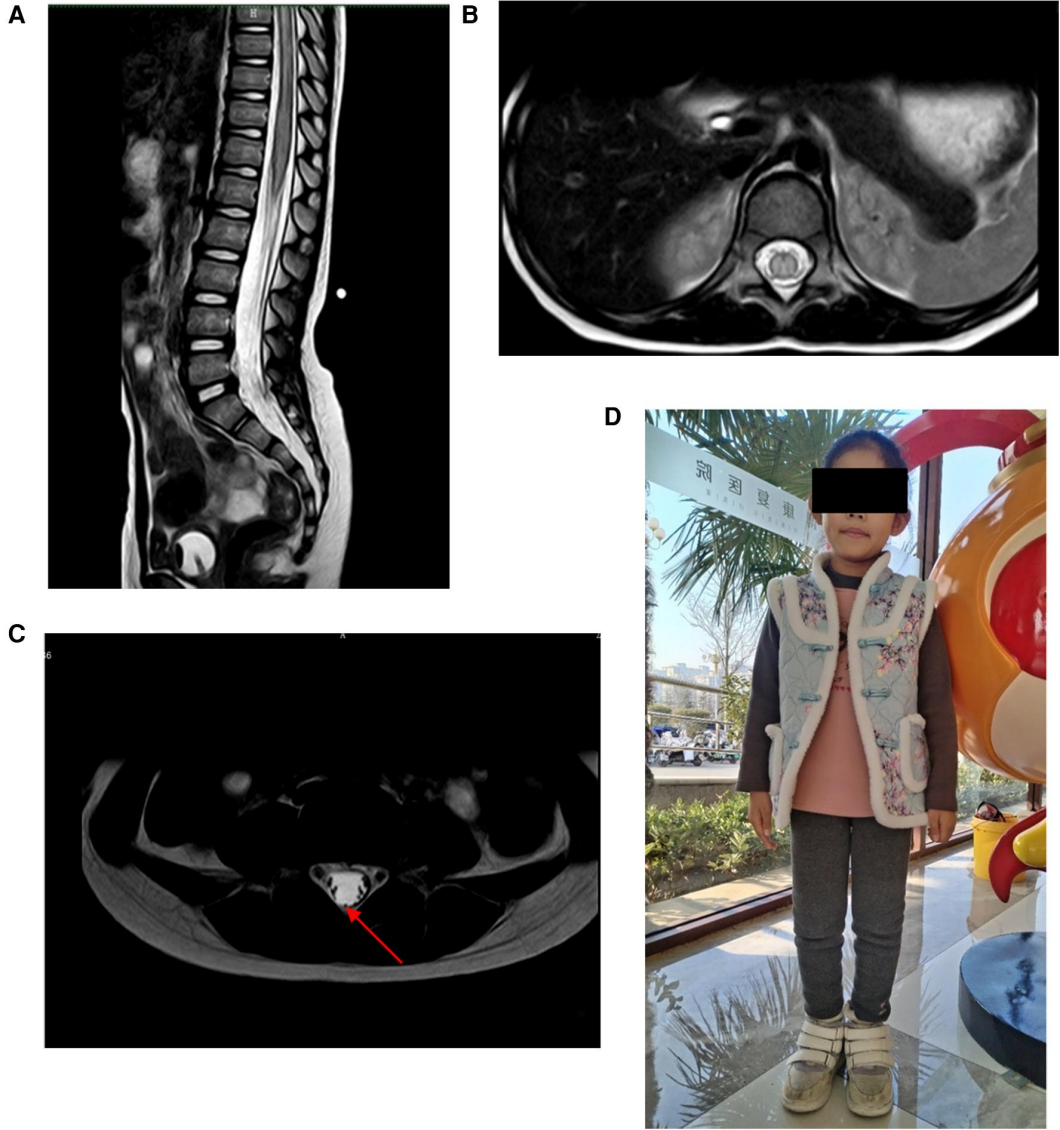
**(A,B)** MRI revealed spinal cord edema. **(C)** MRI revealed thickening of the filum terminale (red arrow). **(D)** At 6 months follow-up, the patient could walk with assistive devices.

Case 1: A 5-year-old girl presented with sudden onset of lower back pain after her lumbar region had been stretched for 29 h prior to admission. Thirty minutes later, she could only crawl, and within an hour after the injury, she lost movement in her lower limbs, accompanied by fecal incontinence and urinary retention. She was urgently transported to our emergency department, where the emergency physician inserted a urinary catheter and promptly requested a neurosurgical consultation. On examination, the patient had normal muscle strength and tone in her upper limbs but grade 0 muscle strength and decreased tone in her lower limbs. She had loss of pain and temperature sensation below the knees bilaterally, with slightly diminished tactile sensation, corresponding to ASIA grade B. Lumbar spine MRI revealed spinal cord edema and thickening of the filum terminale (Figure 1). To prevent further deterioration, she underwent MIIA for intraspinal canal exploration. Intraoperatively, cerebrospinal fluid appeared slightly bloody, and exploration revealed a small arterial bleed, which was irrigated with saline, resulting in the return of normal cerebrospinal fluid color without significant bleeding. Further exploration revealed abnormal thickening and tension of the filum terminale. Surgery concluded after Filum Terminale Transection. Pathological examination revealed fibrous degeneration of the filum terminale. Postoperatively, the patient's condition was stable, with grade 1 muscle strength in bilateral ankle joints and toes, and the urinary catheter remained in place. She was transferred to a rehabilitation hospital for further management. At the 6-month follow-up, the patient had normal bladder and bowel function, could walk with assistive devices, and had grade 4 muscle strength in both lower limbs (Figure 1). Pain, temperature, and tactile sensations were normal in both lower limbs. The patient was classified as ASIA grade D.

**Figure 2:**
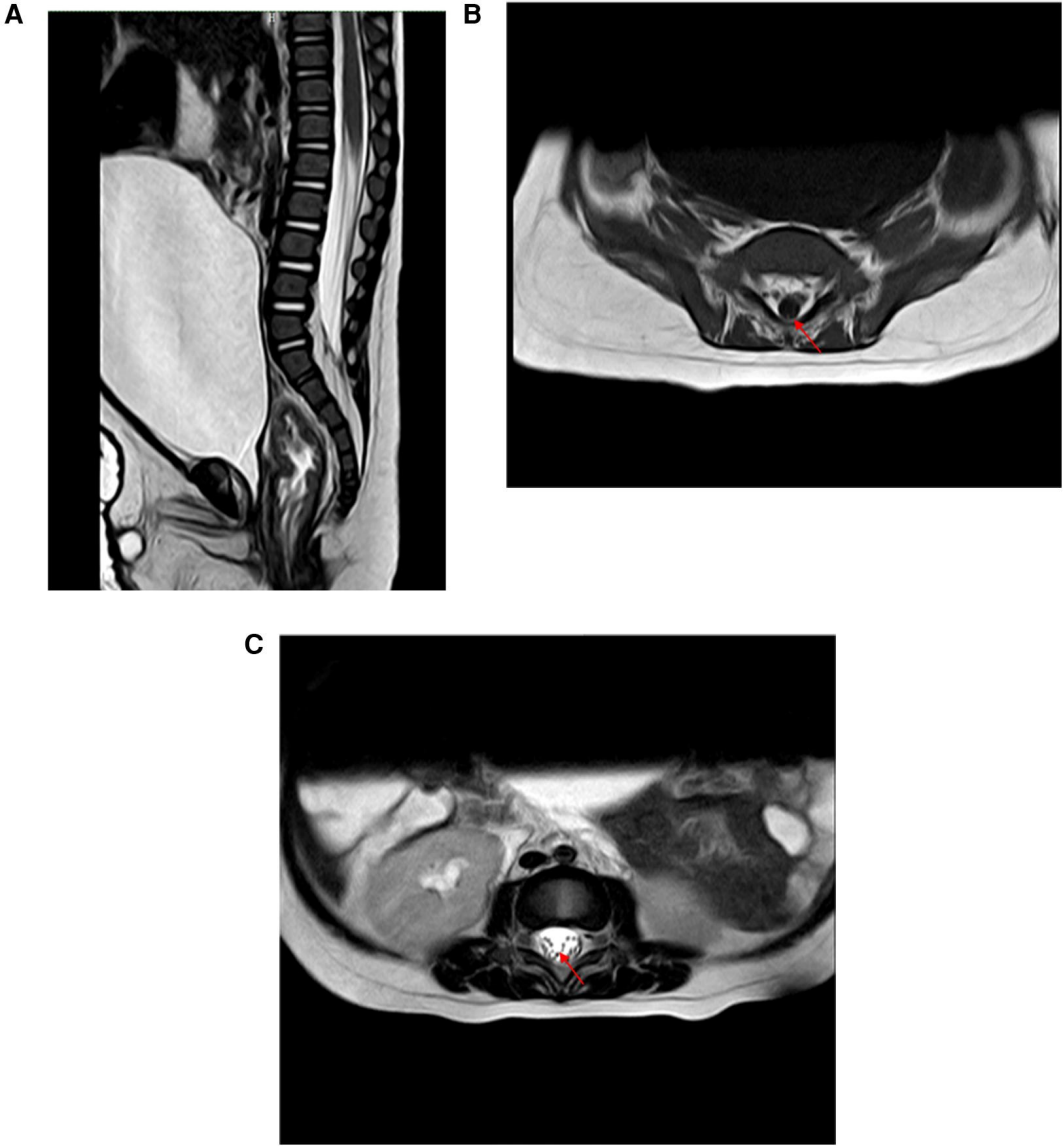
**(A)** MRI revealed bladder distension. **(B,C)** MRI revealed fatty degeneration of the filum terminale (red arrow).

**Figure 3:**
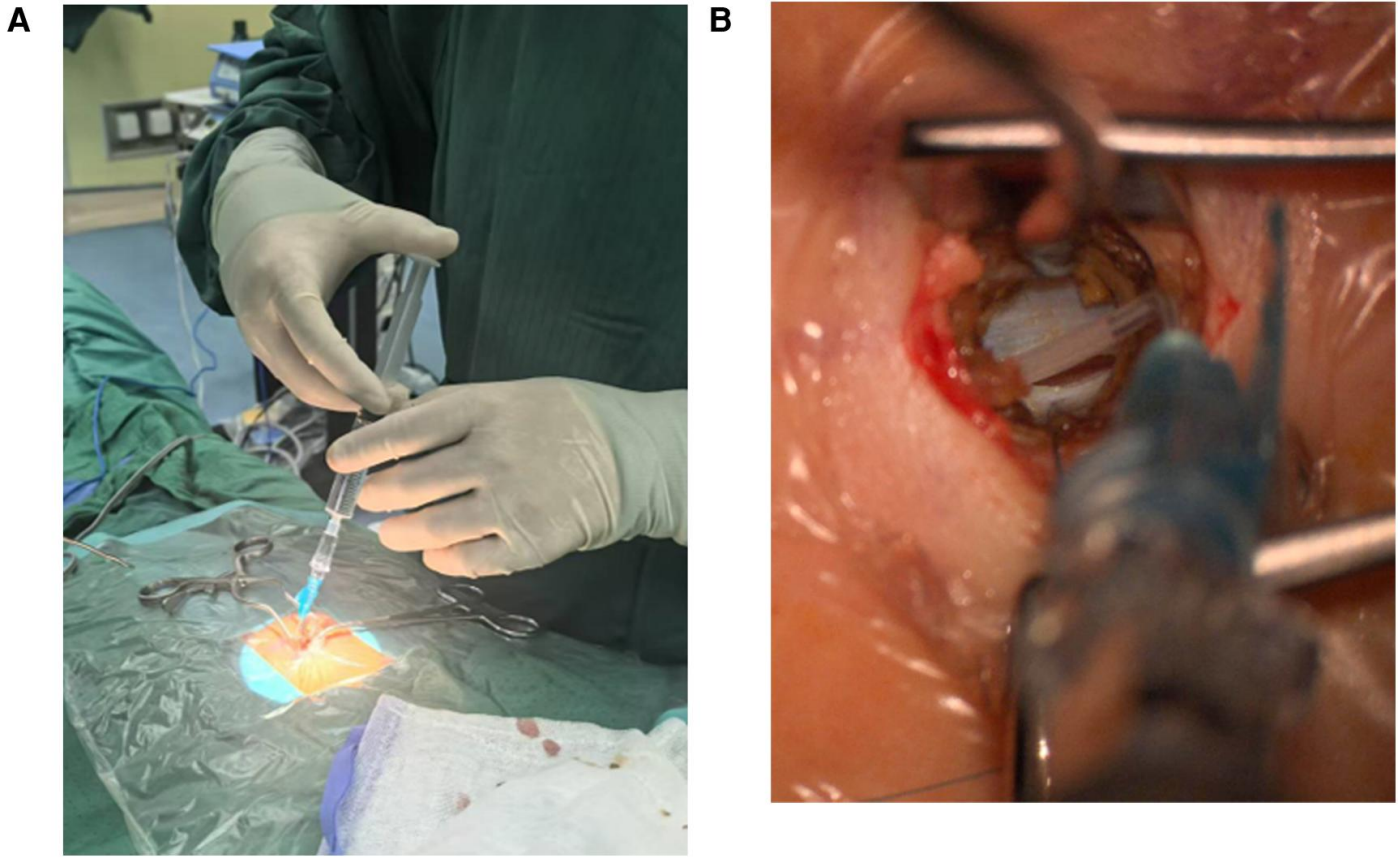
**(A,B)** intraoperatively, the cerebrospinal fluid appeared slightly bloody, which normalized after repeated irrigation with saline.

Case 2: A 7-month-old male infant fell from a sofa at home 10 days before admission. No obvious abnormalities were noted at the time, and medical attention was not sought. Five days later, the infant's parents noticed a significant reduction in movement in the infant's lower limbs. They presented to the local hospital, where x-rays of the lower limbs showed no obvious abnormalities and no treatment was given. On the 7th day after the injury, the infant had no movement in the lower limbs, abdominal distension, and decreased urine output compared to before. Consequently, the infant's parents brought him to our pediatric neurosurgery outpatient clinic. On examination, the infant had normal muscle strength in the upper limbs, abdominal distension, grade 0 muscle strength in the lower limbs, and decreased muscle tone. The infant was classified as ASIA grade B. After admission, MRI of the lumbosacral spine revealed fat-infiltrated filum terminale and bladder distension (Figure 2). A urinary catheter was inserted. To prevent further deterioration, the infant underwent MIIA for intraspinal canal exploration. Intraoperatively, the cerebrospinal fluid appeared slightly bloody, which normalized after repeated irrigation with saline (Figure 3). Severe fatty degeneration and tension of the filum terminale was observed, with significant edema and adhesions of the surrounding cauda equina nerves. Filum terminale transection and disentanglement of the adherent cauda equina nerves were performed before concluding the surgery. Postoperative pathology confirmed fatty degeneration of the filum terminale. The infant's condition remained stable postoperatively, with muscle strength in the lower limbs improving to grade 3 at discharge, and the urinary catheter left in place. At the 2-month follow-up, the infant's urinary function had returned to normal, and limb movements were normal. The infant was classified as ASIA grade E.

**Figure 4:**
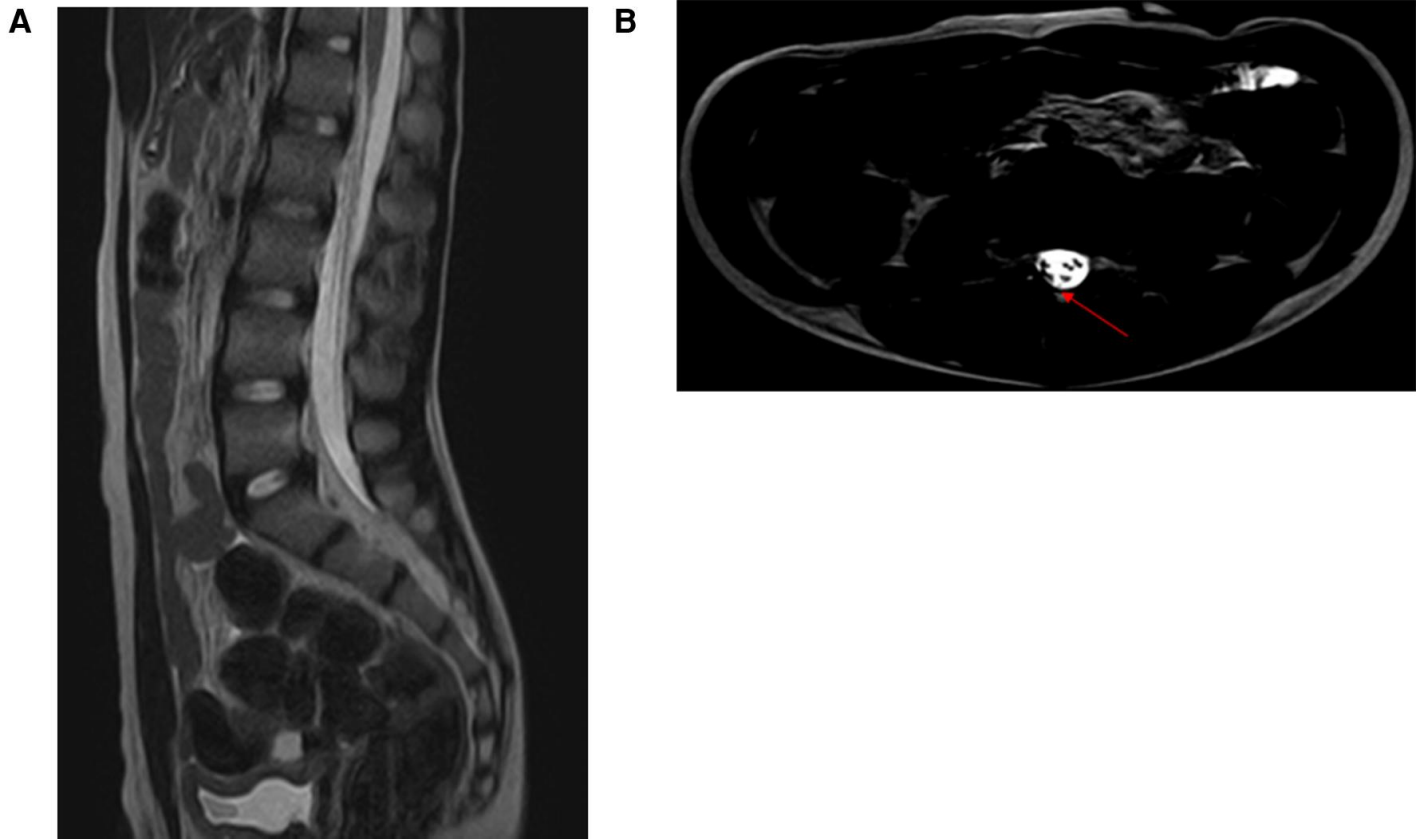
**(A,B)** MRI of the lumbosacral spine in the prone position revealed that the filum terminale was thickened and deviated dorsally, suggesting the possibility of tight filum terminale.

Case 3: An 11-year-old boy with a history of cerebral palsy fell three weeks before admission. Three days later, his parents noticed that he had developed urinary retention and abdominal distension with associated pain. The patient was classified as ASIA grade E. He underwent urinary catheterization at a local hospital, but subsequent attempts to remove the catheter at multiple hospitals were unsuccessful. His parents then sought consultation at our neurosurgery outpatient clinic. MRI of the lumbosacral spine in the prone position revealed that the filum terminale was thickened and deviated dorsally, suggesting the possibility of tight filum terminale (Figure 4). To address the urinary dysfunction, the boy underwent MIIA for intraspinal canal exploration and filum terminale transection. Intraoperatively, the filum terminale was markedly tense, with significant edema of the cauda equina and evident adhesion to the filum terminale. The cerebrospinal fluid appeared slightly bloody, with no apparent vascular bleeding observed during the exploration. Normalization of cerebrospinal fluid color was achieved through repeated irrigation with saline, followed by disentanglement of the adherent cauda equina nerves from the filum terminale. Postoperative pathology confirmed filum terminale fibrous degeneration. The urinary catheter was removed one week postoperatively, and urinary function returned to normal. At the 3-month follow-up, the patient's parents indicated that his previous constipation issues did not recur after surgery. The patient was classified as ASIA grade E.

**Figure 5:**
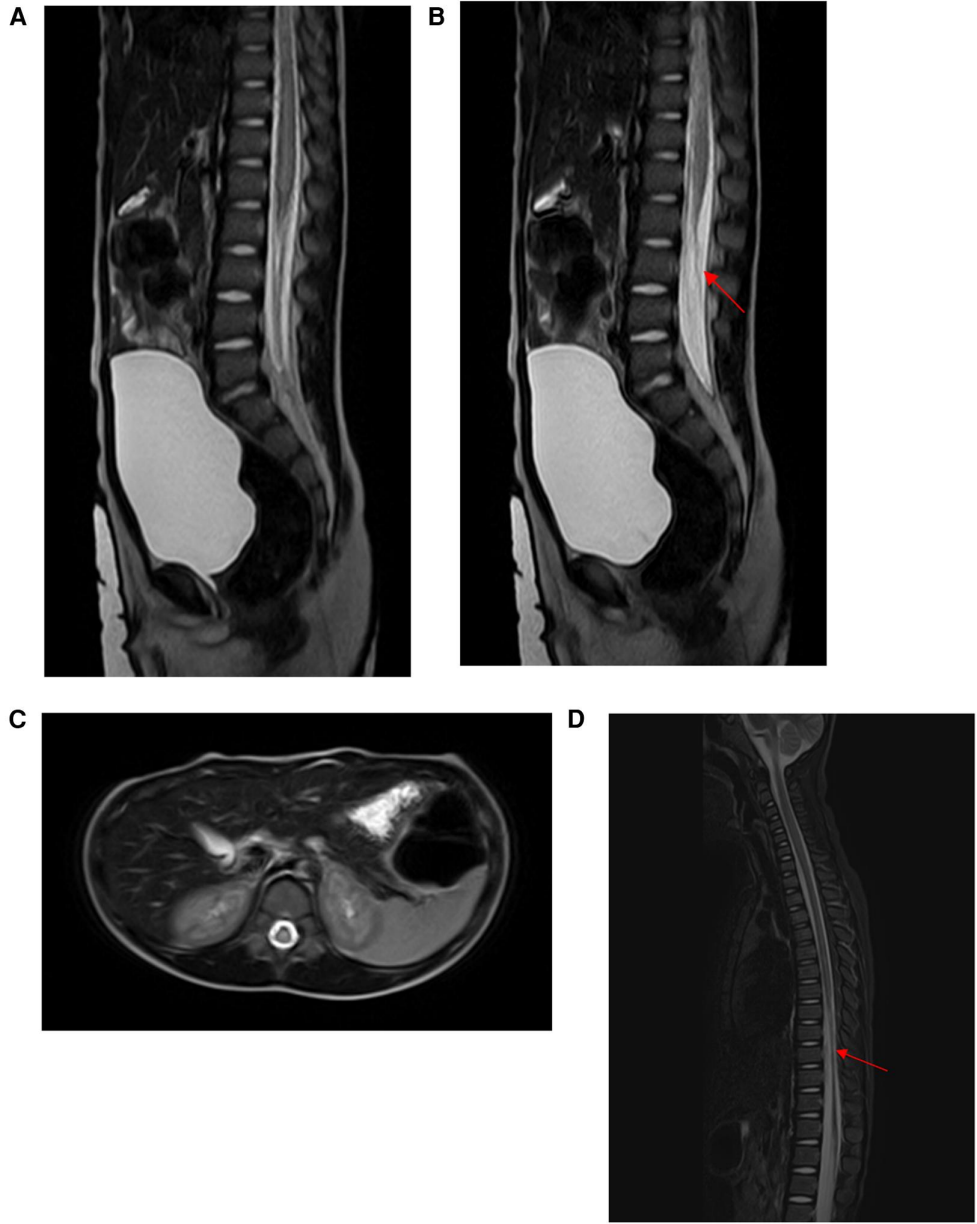
**(A,C)** MRI revealed significant spinal cord edema. **(B)** MRI revealed the filum terminale was thickened and deviated dorsally, suggesting the possibility of tight filum terminale. **(D)** Five months postoperatively, MRI revealed spinal cord atrophy.

Case 4: A 3-year-old boy experienced spinal hyperextension when falling from a sofa onto the floor. Seven hours later, he developed numbness in both lower limbs and bilateral hip regions, which rapidly progressed to paralysis of both lower limbs and loss of sensation below the hips. Fourteen hours post-injury, the boy's parents brought him to our pediatric neurosurgery outpatient clinic. On examination, he exhibited 0-grade muscle strength in both lower limbs, loss of sensation to touch, pain, and temperature below the hips, abdominal distension, and urinary retention. The patient was classified as ASIA grade A. After admission, urinary catheterization was performed. MRI of the lumbosacral spine in the prone position revealed significant spinal cord edema, and the filum terminale was thickened and deviated dorsally, suggesting the possibility of tight filum terminale (Figure 5). To prevent further deterioration, the boy underwent MIIA for intraspinal canal exploration. Intraoperatively, cerebrospinal fluid appeared normal in color without evident vascular bleeding; however, the filum terminale exhibited abnormal tightness. To prevent further spinal cord damage caused by filum terminale traction, filum terminale transection was performed, thereby concluding the surgery. Postoperative pathology confirmed filum terminale fibrous degeneration. Despite continued rehabilitation therapy after discharge, the patient showed no significant improvement in muscle strength or sensation in both lower limbs. At 5 months postoperatively, the patient's muscle strength and sensation remained unchanged, and urinary catheter removal was not feasible. The patient was classified as ASIA grade A. MRI of the thoracolumbar spine revealed spinal cord atrophy (Figure 5).

## Discussion

SCIWORA has a higher incidence in pediatric patients compared to adults, with studies indicating an incidence rate of 19%–34% among children (9). Due to the unique anatomical structure of the pediatric spine, children aged 0–6 years are particularly susceptible to this condition. The etiology of SCIWORA in children is often associated with high-energy injuries, such as those sustained in traffic accidents or sports-related incidents, while SCIWORA resulting from low-energy trauma is relatively rare (4). In contrast, in this case series, all four pediatric patients developed SCIWORA following low-energy trauma and were found to have concurrent TFT. With advancements in imaging technology, SCIWORA may also present with radiological abnormalities, such as spinal cord edema, hemorrhage, or spinal cord transection, as observed on thoracic and lumbar MRI scans. Additionally, TFT, filum thickening, and filum terminale lipoma may be incidentally detected (10).

Given that all four patients in this case series showed evidence of TFT following low-energy trauma, it is hypothesized that TFT may be a precipitating factor for the development of SCIWORA in children after low-energy trauma. The proposed mechanism is that the TFT exerts abnormal longitudinal traction on the conus medullaris and spinal cord, leading to a metabolic disorder similar to ischemic injury (11, 12). Furthermore, the pediatric spine is characterized by loose ligamentous structures around the vertebral bodies, high flexibility, shallow joint surfaces, and greater susceptibility to slippage under external forces (13, 14). The spinal cord has less compliance compared to the spine, and the T4-9 vertebral canal has the narrowest sagittal and transverse diameters, with the fewest anastomoses between intramedullary and extramedullary arteries (4). Even minor traction can easily lead to ischemic necrosis of the spinal cord. In the context of longitudinal traction from the TFT and the anatomical predisposition of the pediatric spine to deformation and ischemic injury, even low-energy trauma may more readily result in SCIWORA in children. Therefore, TFT may be a contributing factor in the development of SCIWORA in children following low-energy trauma.

In recent years, the MIIA for filum terminale transection has been widely reported in the literature for treating TCS caused by filum terminale lipoma or tight filum terminale (15–17). Traditional filum terminale transection typically requires laminectomy to expose the dural, which involves extensive soft tissue dissection, resulting in bony structural damage and an increased risk of postoperative complications such as cerebrospinal fluid leakage (18). In contrast, our MIIA technique involves a small, approximately 1.5 cm midline incision between L3 and L4, providing a minimal surgical corridor through the interlaminar space. This approach allows for minimal muscle dissection and a small durotomy, achieving minimal arachnoid dissection without bony structural damage (19). Compared to traditional methods, MIIA offers significant advantages in preventing retethering and ensuring watertight dural closure. Relevant literature indicates that, while ensuring filum transection, MIIA has smaller skin incisions, less soft tissue injury, milder postoperative pain, less blood loss, and fewer intradural procedures compared to traditional transection, thereby effectively reducing the risk of retethering and cerebrospinal fluid leakage (18–20). Moreover, patients can mobilize earlier postoperatively, which is particularly beneficial for pediatric patients. In our case series, four patients experienced minimal postoperative pain, short hospital stays, and no impact on subsequent rehabilitation. During follow-up, no patients exhibited retethering or additional neurological symptoms. However, the limited surgical corridor through the interlaminar space may pose challenges in cases with large fatty filum terminale. Notably, none of the four patients in our series had large fatty filum terminale. Overall, MIIA offers the advantages of minimally invasiveness, reduced potential for complications, and decreased risk of retethering.

Currently, there are very few reports on the use of MIIA for filum terminale transection in treating pediatric patients with SCIWORA and concurrent TFT. In this case series, all four patients underwent MIIA for filum terminale transection and intraspinal canal exploration due to progressive neurological impairment. Postoperatively, none of the patients experienced further neurological deterioration. Two patients experienced complete resolution of preoperative symptoms within three months post-surgery, one patient showed significant neurological improvement, and one patient did not experience improvement in preoperative symptoms. The patient whose symptoms did not improve before surgery had a preoperative ASIA score of Grade A, indicating a significantly higher degree of neurological damage compared to the other three patients. This suggests a stronger irreversibility of neurological damage. Moreover, preoperative MRI revealed more pronounced spinal cord edema than in the other cases. It is speculated that these two factors may be the primary reasons for the patient's lack of postoperative neurological improvement and the development of spinal cord atrophy. None of the patients developed postoperative complications such as cerebrospinal fluid leakage, poor wound healing, or wound infection. The potential effectiveness of filum terminale transection in treating pediatric patients with SCIWORA and concurrent TFT may be attributed to the relief of abnormal longitudinal traction exerted by the inelastic filum terminale (21). This traction causes oxidative metabolic impairment in the central nervous system and places the spinal cord in a state of mild to severe ischemia-hypoxia. Whether this state is reversible depends on the extent and duration of the damage. In cases where SCIWORA occurs in patients with TFT, the injured spinal cord continues to be subjected to longitudinal traction from the inelastic filum terminale, maintaining a state of ischemia-hypoxia that hinders recovery of the damaged nervous system (11). Moreover, prolonged and severe traction on the spinal cord can result in irreversible neurological damage. Therefore, early filum terminale transection after the onset of SCIWORA in patients with TFT may help alleviate the ischemia-hypoxia state caused by spinal cord traction, potentially facilitating recovery of the already damaged nervous system.

## Conclusions

This study described the treatment outcomes of four children with SCIWORA complicated by tight filum terminale after undergoing filum terminale transection via MIIA. All the children suffered from low-energy injuries, and no complications occurred after the operation. The neurological impairment symptoms of three children were significantly improved, and the neurological impairment symptoms of one child did not further aggravate. Therefore, we inferred that tight filum terminale might be the etiology of SCIWORA in children after suffering from low-energy injuries. Performing filum terminale transection as early as possible after the occurrence of SCIWORA complicated by tight filum terminale in children might be beneficial for relieving the state of spinal cord ischemia and hypoxia caused by longitudinal traction of the spinal cord as early as possible and facilitating the recovery of neurological injuries. Due to the small sample size of this study, the conclusions are preliminary and exploratory. Future studies will expand the sample size to further investigate and validate the reliability and generalizability of the findings.

## Data Availability

The original contributions presented in the study are included in the article/Supplementary Material, further inquiries can be directed to the corresponding authors.
